# Batch fabrication process of biomimetic wing with high flexibility of stiffness design for flapping-wing micro aerial vehicles

**DOI:** 10.1016/j.mex.2020.101121

**Published:** 2020-10-29

**Authors:** Takashi Ozaki, Kanae Hamaguchi

**Affiliations:** Toyota Central R&D Labs. Inc., Japan

**Keywords:** Biomimetic flapping wing, Hot press, Passive pitching

## Abstract

A lamination-based batch-fabrication process of biomimetic wing for flapping-wing micro aerial vehicles is presented. The key benefits of this process are:

• One of the advantages of the process is high productivity; eight wings were successfully fabricated simultaneously in our experiment.
• The wing fabricated with the reported process is made of soft polyimide and partially reinforced by a titanium layer. This configuration enables the flexible design of the bending stiffness distribution on the wing, which is the key specification for generating lift force.
• The reinforcing material can be replaced with other metals or heat-resistant polymers, and the number of layers and layer thicknesses are also variable. This indicates that the reported process can be customized considerably to suit individual needs.

## Graphical abstract

**Figure:**
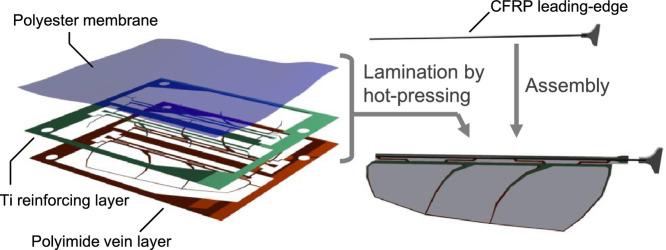

**Table:** Specifications table

Subject Area:	Engineering
More specific subject area:	*Micro aerial vehicles*
Method name:	*Batch fabrication process of biomimetic wing based on hot pressing*
Name and reference of original method:	*There are no original methods.*
Resource availability:	*There are no resources linked with this manuscript.*

## Background

Flapping-wing micro aerial vehicles (FWMAVs) have gained attention for their high maneuverability and efficiency, inherent in natural insects and birds. One of the most important components of an FWMAV is the wing because the generated lift force highly depends on the wing specifications. Many insects and insect-mimetic MAVs generate the lift force by a passive pitching mechanism in steady flight; the wing is passively pitched (deformed) owing to the aerodynamic pressure and wing inertia during wing stroke motion [1,2]. To realize the passive pitching, the wing should be easier to bend in the pitching direction than the wing span direction. It means that design capability in terms of the bending stiffness is required for a wing in FWMAVs. Thus, a fabrication process with high flexibility of stiffness design is demanded. In addition, productivity is also important for the fabrication process.

Previously reported wing-fabrication processes can be mainly classified into three categories: hand-assembly [3,4], casting [5,6], and lamination [7,8]. Our presented protocol is based on lamination, taking into account its high productivity. Some reports by a research group at Harvard University have presented a wing composed of a carbon fiber reinforced plastic (CFRP) vein layer and a polyester membrane [7,8]. They demonstrated that many wings could be fabricated simultaneously with their protocol. However, their wing acts as a rigid plate and the pitching function is realized by an additional hinge assembled at the wing root. In other words, their method does not offer the design capability in terms of bending stiffness. From this background, we developed a lamination-based fabrication process with higher flexibility of the bending stiffness design. In this report, we present a wing design capable of pitching with the fabrication process step by step.

## Structure and materials

**Fig. 1:**
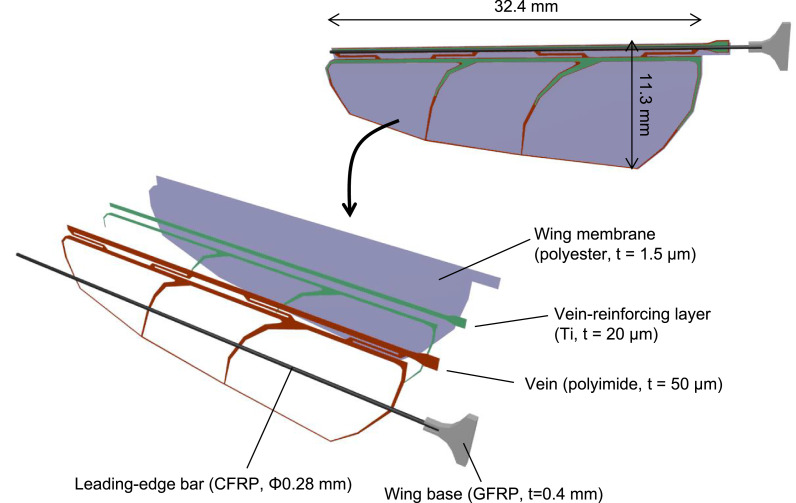
Structure of biomimetic wing.

**Fig. 2:**
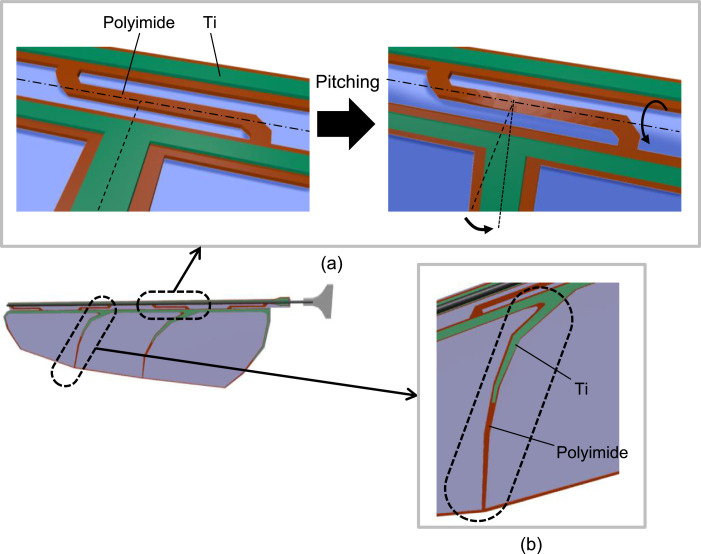
Vein design to realize passive pitching: (a) vein design near the leading edge to realize pitching-hinge functionality (not to scale), and (b) vein extending along the wing chord to keep the wing surface flat.

The wing structure presented in this report is shown in Fig. 1. The wing is composed of titanium and polyimide composite veins, polyester membrane, a CFRP leading-edge bar, and a glass fiber reinforced plastic (GFRP) base. Among these components, the design of the Ti/polyimide veins is the most important for the lift force generation because it determines the stiffness distribution of the wing. The presented wing was designed to bend sharply near the upper edge in the direction of the pitching and to keep the other area as flat as possible. Specifically, the veins near the upper edge are made of a polyimide single layer and run along the wing span direction, as shown in Fig. 2a, so that this region becomes soft and works as a pitching hinge. On the other area of the wing, veins extend along the wing chord. In addition, these veins are partially reinforced with a titanium layer to increase the bending stiffness, as shown in Fig. 2b. A key material of this structure is the polyimide layer. We employed “heat bondable” polyimide; this was originally used for manufacturing flexible printed circuits and can be bonded to metals by heat-pressing. This material enables partial reinforcement by the titanium layer, so the flexible design of the wing stiffness become possible. In our experiments, we employed UPILEX-VT (Ube Industries, Ltd, Tokyo, Japan) with a thickness of 50 µm for the heat-bondable polyimide. The thicknesses of the titanium layer and the polyester membrane are 20 µm and 1.5 µm, respectively. The diameter of the CFRP rod is 0.28 mm.

## Fabrication process

**Fig. 3:**
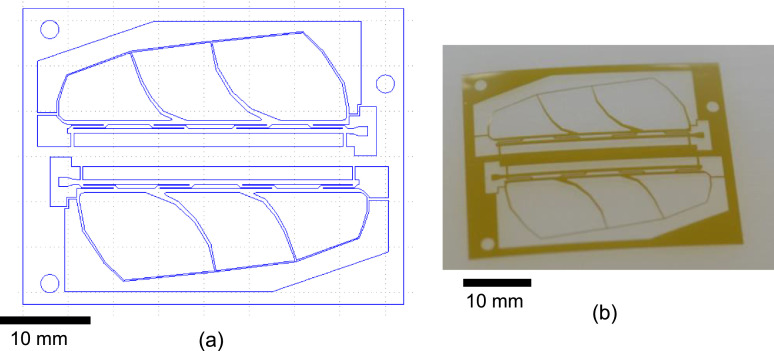
Polyimide vein layer: (a) CAD layout for laser cutting, and (b) photograph of fabricated specimen.

**Fig. 4:**
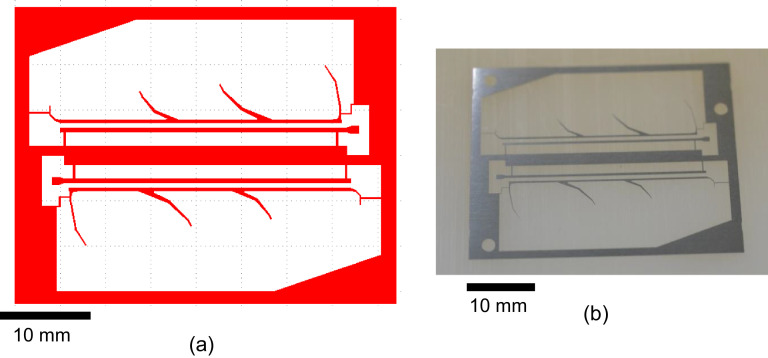
Ti vein layer: (a) CAD layout for wet-etching, and (b) photograph of fabricated specimen.

**Fig. 5:**
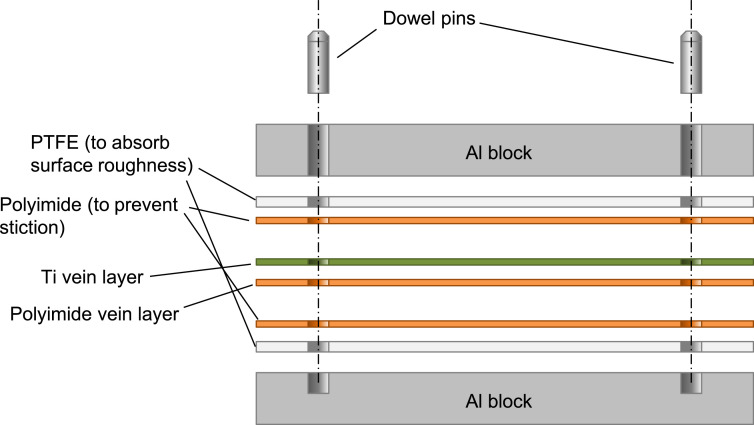
Setup of hot-press for bonding polyimide and Ti layers (not to scale).

**Fig. 6:**
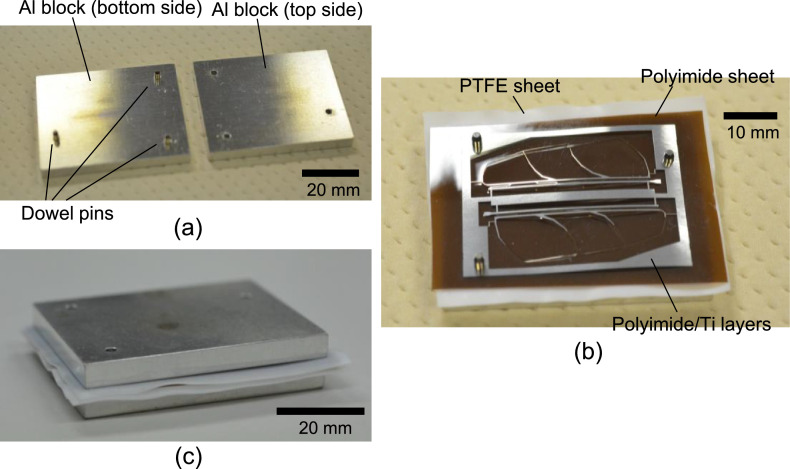
Photograph of hot-press setup: (a) Al alignment blocks, (b) polyimide and Ti layers set on Al block, and (c) specimen before hot-pressing.

**Fig. 7:**
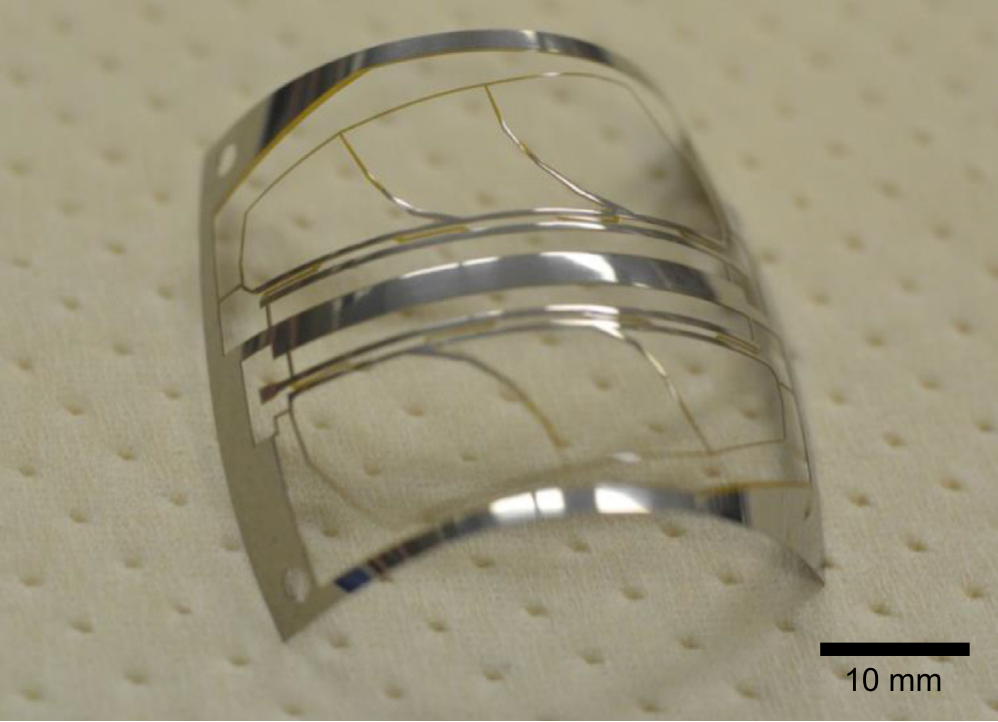
Laminated polyimide/Ti vein layer after hot-pressing.

**Fig. 8:**
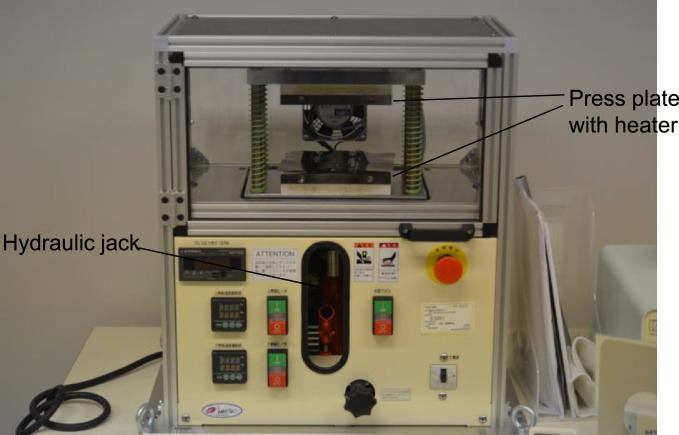
Hot-pressing machine.

The polyimide layer and Ti layer were patterned by picoseconds laser processing and wet-etching, respectively. These processes were carried out by Nano-Process. Co. ltd. (Hamamatsu, Japan) and Hirai Seimitsu Kogyo Co., Ltd. (Osaka, Japan) in our experiments. The shapes of these layers are shown in Figs. 3a and 4a. Figs. 3b and 4b show photographs of the fabricated polyimide and Ti sheets, respectively. In our design, the layouts of two wing patterns are realized on a sheet. The wing patterns are suspended on the outer frame by some thin beams. On the outer frame, three holes for dowel pins are placed. Fig. 5 illustrates the setup of the hot-pressing. The polyimide and Ti sheets are stacked and sandwiched by other polyimide sheets, polytetrafluoroethylene (PTFE) sheets, and aluminum (Al) blocks. These additional polyimide sheets are not heat bondable, and they prevent the heat-bondable polyimide sheet from adhering to undesired parts. The PTFE sheets are used to absorb the surface roughness and inclination in the pressing process. We employed UPILEX-S (Ube Industries, Ltd, Tokyo, Japan) with a thickness of 75 µm for the additional polyimide sheets, and Nafron (NICHIAS Corporation, Tokyo, Japan) with a thickness of 1 mm for the PTFE sheets. On an Al block, three dowel pins (with the diameter of 2 mm) are erected as shown in Fig. 6a, and with these pins the polyimide and Ti sheets are aligned. Fig. 6b shows the polyimide and Ti sheets set on the Al plate, and Fig. 6c shows the specimen ready for the pressing process. The pressure and temperature are 0.5 MPa and 310 °C, respectively. We maintained the temperature for 10 min and then allowed it to cool down. After the specimen was cooled to the room temperature, it is ejected. Fig. 7 shows a photograph of the laminated polyimide/Ti layers. The specimen usually warps due to thermal stress between the polyimide and Ti layers. If this is undesired for the intended use, symmetric configurations, i.e., polyimide/Ti/polyimide or Ti/polyimide/Ti, are suitable. We employed a hot-pressing machine (IMC-180C) produced by Imoto Machinery Co., Ltd. (Kyoto, Japan), as shown in Fig. 8. We processed four sets of the setup (Fig. 6c) simultaneously; eight wings were fabricated at a time. Note that the number of the wings in a batch is limited by the specifications of the pressing tool; if we increase the table size and pressing force, more wings can be processed in a batch.

**Fig. 9:**
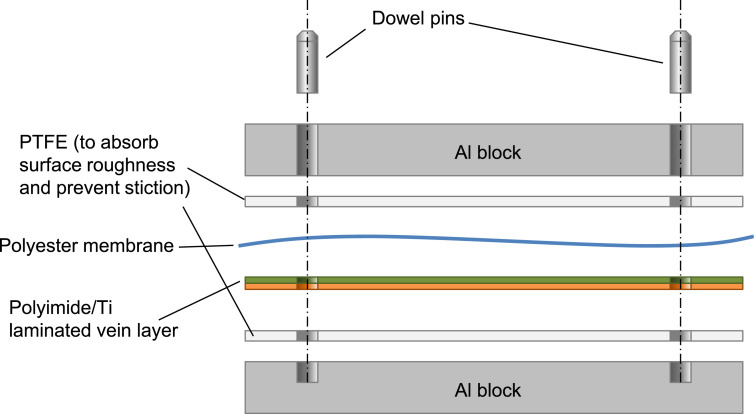
Setup of hot-press for laminating polyester membrane (not to scale).

**Fig. 10:**
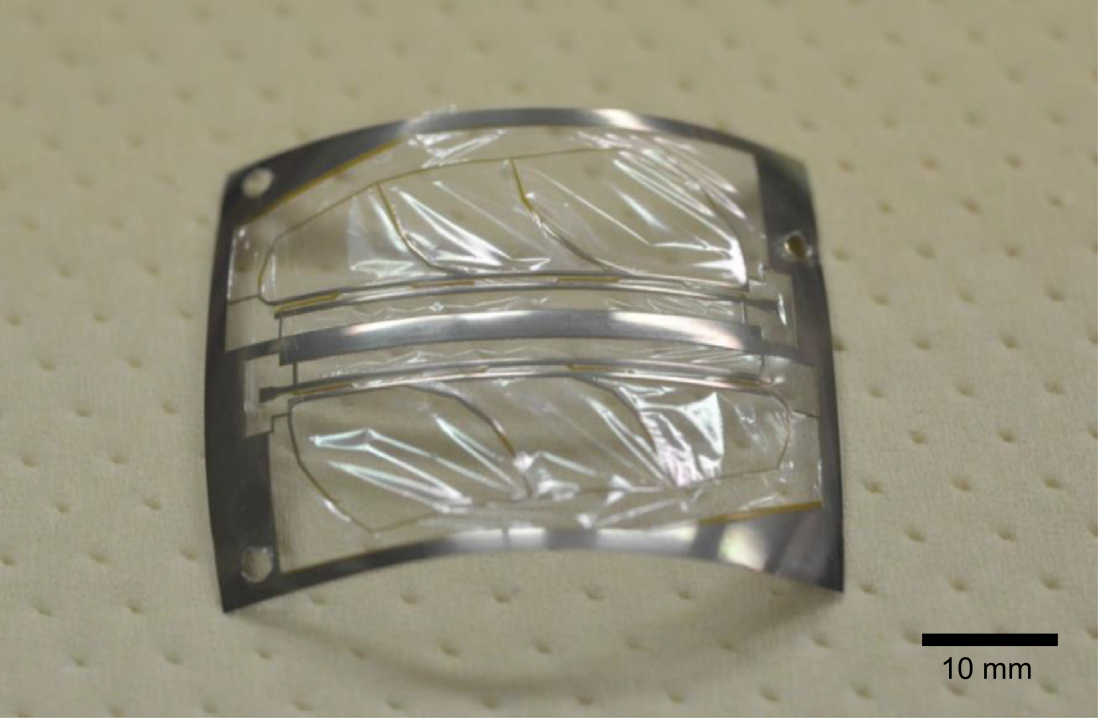
Photograph of specimen with polyester membrane.

Next, the polyester film is laminated on the polyimide/Ti structure. The press pressure and temperature are 0.5 MPa and 220 °C, respectively. In this step, when the temperature reaches 220 °C, we turn off the heater immediately; long heating time will result in breakage of the polyester film. The pressing setup for this step is shown in Fig. 9. The polyimide sheet for preventing adhesion is not used in this step because it does not work for the polyester film; the polyester film sticks to polyimide by heating. We used Ultra-Polyester (Chemplex Industries, Inc., Florida, United States) for the membrane. The polyester is not patterned and just overlaid on the Ti/polyimide structure. Because the membrane is very thin, it can be easily penetrated by the dowel pins. In our experiments, we processed four units simultaneously, the same as the previous step. When the specimen was cooled near room temperature, it was removed from the press machine and disassembled. Fig. 10 shows a photograph of the specimen after the polyester lamination. The polyester membrane may be difficult to peel off from the PTFE sheet. In such a case, immersing the specimens in ethanol is effective in separating them.

**Fig. 11:**
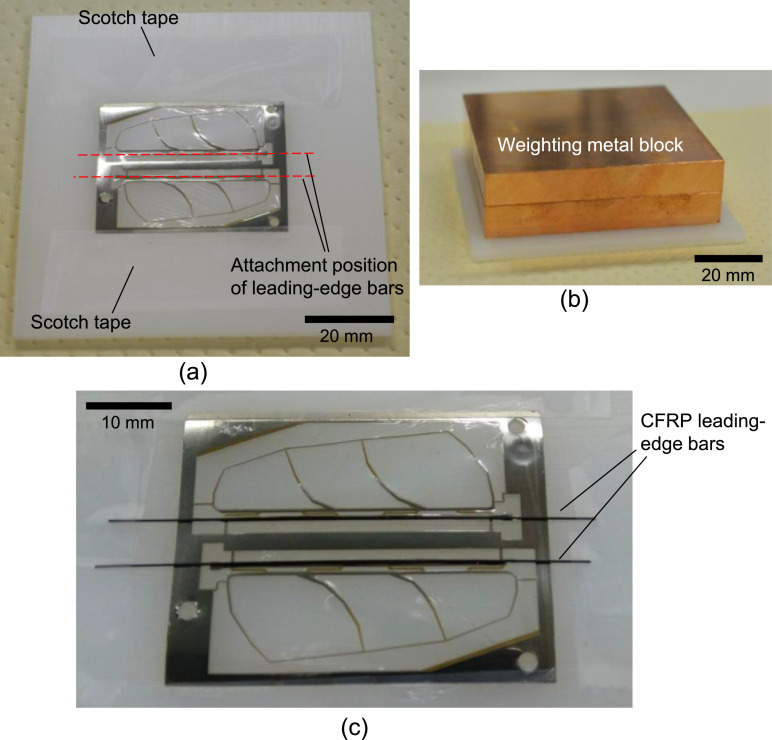
Procedure of bonding leading-edge bar: (a) attachment position on wing surface, (b) weighting setup for epoxy adhesive curing, and (c) photograph of specimen after the bonding.

The specimen is fixed on a plastic substrate by Scotch tape as shown in Fig. 11a. A CFRP rod (vDijk Pultrusion Products, Tilburg, Netherlands) was cut into the desired length, and then coated with epoxy adhesive (Quick 5; Konishi Co., Ltd., Osaka, Japan). The rod was placed on the leading edge of the wings, which is indicated by the dotted lines in Fig. 11a. A PTFE sheet with a thickness of 1 mm was put on the specimen and they were weighted by a metal block (with a weight of approximately 440 g), as shown in Fig. 11b, which was to ensure the contact between the CFRP rod and the wing layers. The adhesive was cured for at least 6 h. After the epoxy adhesive was cured, we removed the weight and took the specimen out. Fig. 11c shows a photograph of the specimen after the leading-edge bar bonding.

**Fig. 12:**
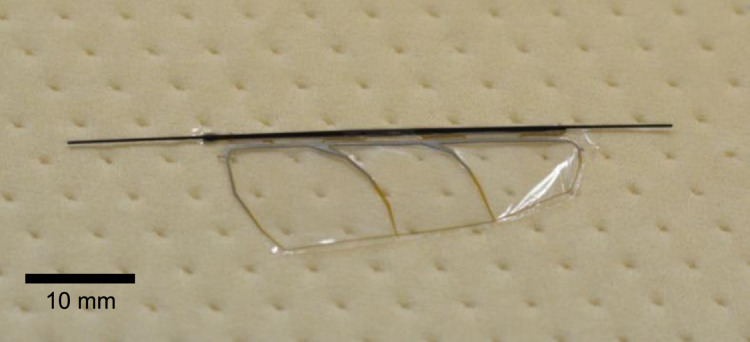
Specimen after frame removal.

**Fig. 13:**
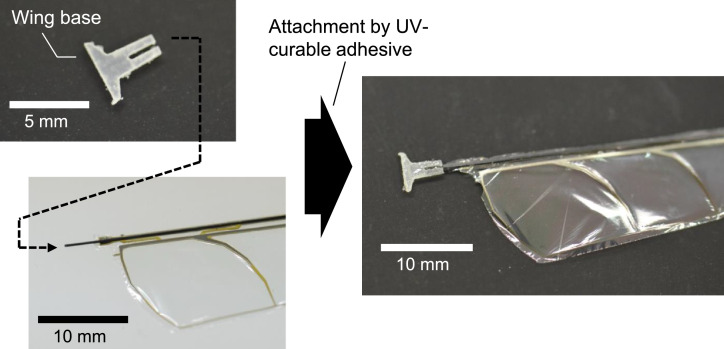
Attachment of fixture part at wing root.

**Fig. 14:**
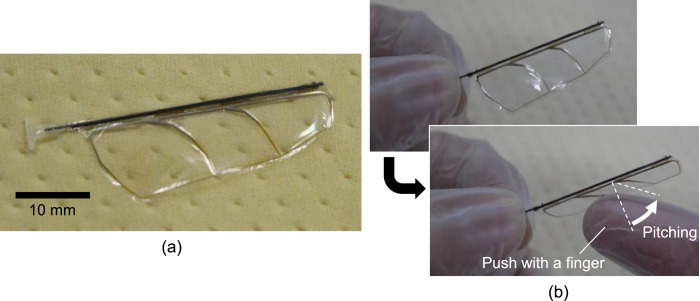
Completed wing: (a) appearance photograph and (b) pitching demonstration by pushing with a finger.

Next, the polyester film was cut along the wing outline. We used a knife for this cutting. If the number of the wings is not large, cutting by hand with a knife is apparently the easiest and fastest way. Another way is laser processing. We tried the cutting process with a CO_2_ laser instrument (Epilog Mini 18; Epilog Laser, Colorado, United States), and found that it also gave good results. After the membrane cutting, the suspension beams were cut with nippers to separate the wing structure and the frame. Fig. 12 shows a photograph of the specimen after the removal of the frame. Then, both ends of the CFRP rod were cut to the desired lengths. A GFRP base part was attached at the root of the CFRP rod by UV-curable adhesive (Loctite 4305; Henkel AG & Company, Düsseldorf, Germany), as shown in Fig. 13. This part is for fixing the wing on our flapping actuator, so it is optional, depending on the individual fixture designs. We fabricated this part from a GFRP plate (FS-010) purchased from APC Composites. Inc. (California, United States). Fig. 14a shows a photograph of a finished wing. Fig. 14b shows the pitching functionality; the wing bends in the direction of pitching when it is pushed with a finger. We have also attached a video file, which is the source of Fig. 14b. It can be found that the wing is flexible about the pitching deformation and rigid in the wing span direction in accordance with the design concept.

## Conclusion

We demonstrated batch fabrication of biomimetic wings for micro aerial vehicles. Owing to partial reinforcement with the Ti layer of the wing veins, a highly flexible design of the wing bending stiffness becomes possible with the reported process. In addition, because it is based on a layer-by-layer lamination process, high productivity is also achieved; in the experiment, we fabricated eight wings in a batch and it can be easily increased by increasing the size of the hot-pressing machine. The reported method is not limited to the Ti/polyimide structure; the Ti layer could be replaced with other metals and heat-resistant polymers. A multilayered structure, such as Ti/polyimide/Ti composite, should be also possible because the heat-bondable layer is coated on both sides of the polyimide. This indicates that there is considerable room for the customization of the structure to suit different individual vehicle designs.

## Declaration of Competing Interest

The Authors confirm that there are no conflicts of interest.

## Appendix Supplementary materials

Image, video 1

## References

[1] Ennos A.R. (1988). The inertial cause of wing rotation in diptera. J. Exp. Biol..

[2] Bergou A.J., Xu S., Wang Z.J. (2007). Passive wing pitch reversal in insect flight. J. Fuild Mech..

[3] Lentink D., Jongerius S.R., Bradshaw N.L., Floreano D, Zufferey JC, Srinivasan M, Ellington C (2009). The scalable design of flapping micro-air vehicles inspired by insect flight. Flying Insects and Robots.

[4] Nan Y., Karásek M., Lalami M.E., Preumont A. (2017). Experimental optimization of wing shape for a hummingbird-like flapping wing micro air vehicle. Bioinspir. Biomim..

[5] Tanaka H., Matsumoto K., Shimoyama I. (2007). Fabrication of a three-dimensional insect-wing model by micromolding of thermosetting resin with a thin elastomeric mold. J. Micromech. Microeng..

[6] Tanaka H., Whitney J.P., Wood R.J. (2011). Effect of flexural and torsional wing flexibility on lift generation in hoverfly flight. Integr. Comp. Biol..

[7] Wood R.J. (2008). The first takeoff of a biologically inspired at-scale robotic insect. IEEE Trans. Robot..

[8] Ma K.Y., Chirarattananon P., Wood R.J. (2015). Design and fabrication of an insect-scale flying robot for control autonomy. Proceedings of IEEE/RSJ International Conference on Intelligent Robots and Systems.

